# Predictors of Dental Caries Increment in Schoolchildren: A Longitudinal Study of Salivary and Behavioral Risk Factors

**DOI:** 10.3390/dj14060382

**Published:** 2026-06-19

**Authors:** Leonor Sánchez-Pérez, Laura Patricia Sáenz Martínez, Nelly Molina Frechero, Marco Antonio Zepeda-Zepeda, María Esther Irigoyen-Camacho

**Affiliations:** Health Care Department, Biological and Health Sciences Division, Autonomous Metropolitan University (UAM), Mexico City 04960, Mexico; lpsaenz@correo.xoc.uam.mx (L.P.S.M.); nmolina@correo.xoc.uam.mx (N.M.F.); mzepeda@correo.xoc.uam.mx (M.A.Z.-Z.)

**Keywords:** caries, oral hygiene, salivary flow rates, bacterial counts, buffer capacity, dental caries increment

## Abstract

**Background:** This study analyzed the association between caries increment and clinical, salivary, bacteriological, and behavioral risk markers in a two-year follow-up study of schoolchildren in Mexico City. **Methods:** A two-year follow-up study was conducted in elementary schoolchildren, where 118 schoolchildren aged 7–10 years at baseline (50% boys) participated in the follow-up. Toothbrushing frequency, sugar consumption, and dental caries indices were recorded according to WHO criteria. Salivary secretion rates, buffering capacity (Dentobuff^®^), and cariogenic bacterial counts (Dentocult SM and LB^®^) were also measured. Logistic regression was applied to analyze associations between caries increment and risk markers. **Results:** The mean baseline caries indices were dmft 4.8 (SD 4.0) and DMFT 0.6 (SD 0.9). Children were classified into three caries experience groups: caries-free, filled-teeth, and caries-active. After two years, baseline caries-free children had a lower caries increment in permanent teeth (0.2, SD 0.7) than other groups (*p* < 0.0001). However, the caries increment was similar between groups (*p* = 0.0827). Logistic regression revealed associations with toothbrushing frequency [OR = 2.77, *p* = 0.026], *S. mutans* counts [OR = 3.38, *p* = 0.050], and *Lactobacillus* counts [OR = 2.91, *p* = 0.029]. **Conclusions**: Children with low toothbrushing frequency and high cariogenic bacterial counts developed more caries lesions than those with better oral hygiene and lower bacterial levels. Greater emphasis should be placed on promoting oral hygiene and reducing bacterial load in the oral cavity.

## 1. Introduction

Caries, the most common chronic disease among children and adults, is a multifactorial disease with diverse risk factors, for which the biological, behavioral, and lifestyle determinants remain to be fully determined. Untreated dental caries and tooth loss are prevalent globally, with wide variations among countries, age groups, and socioeconomic statuses [1].

Oral diseases affect up to 90% of the global population, with dental caries being the most common condition in childhood. According to 2024 data from Mexico’s Ministry of Health epidemiological surveillance system (SIVEPAB), more than 62% of patients present moderate or poor oral hygiene. In addition, 70% of children aged 6–12 have at least one caries lesion, and increment rates remain high [2].

Caries prevalence has a highly skewed distribution, suggesting the need to develop new and effective preventive or therapeutic perspective approaches, especially for high-risk groups. Caries risk assessment still has great potential to improve patient care, as it is the cornerstone of a minimally invasive care plan, allowing appropriate non-invasive and invasive interventions and recovery strategies such as follow-up salt or water fluoridation, topical fluoride application, the use of fluoride rinses, an emphasis on toothbrushing with a fluoride dentifrice, flossing, a proper sugar intake, and regular dental office visits, among other clinical strategies [3].

*Streptococcus mutans* and some species of *Lactobacillus* have been implicated in tooth decay, along with several other species of the oral microbiome. In this context, dental biofilm is a critical factor in the multifactorial etiology of caries, but it does not determine the disease on its own. The salivary flow rate can affect biofilm formation [4], as reduced salivary secretion rate can increase bacterial adherence to the tooth surface. In addition, oral hygiene is the most economical and effective practice for maintaining oral health [5].

There are currently a wide variety of methods for identifying caries risk; some focus on clinical factors, and others on microbiological or nutritional factors. In addition, they consider sex, socioeconomic status, and maternal health, and suggest considering the different ways in which each population develops the disease.

The evaluation of caries risk has focused on the analysis of bacteriological, salivary, and clinical markers that can be used as risk predictors. The most used markers are *S. mutans* and *Lactobacillus* counts, salivary secretion rate, caries experience, and sugar intake frequency [6].

The aim of this study was to analyze the association between caries increment and clinical, salivary, bacteriological, and behavioral risk markers in a two-year follow-up study of schoolchildren in Mexico City.

## 2. Materials and Methods

### 2.1. Ethics

A two-year follow-up cohort study was performed. This project was approved by the Research Commission at the Universidad Autónoma Metropolitana (DCBS 10/17; 4/25) and by the Ethics Committee (IRB: CE.002.17; CEI.2025.006.). Parents were informed of the results of their children’s oral health status on two occasions. After the initial examination, parents were advised about institutions that could provide dental and medical services in their community. All children receive oral health education sessions and new toothbrushes at six-month intervals, free of charge.

### 2.2. Location

This study was carried out in a public (state-funded) school in Mexico City. Based on security, education, employment, and housing quality, the area is classified as middle-income, in accordance with the classification developed by Mexico’s National Institute of Geography and Informatics, using these indicators at the neighborhood level. Drinking water in this area contains <0.25 ppm fluoride. The table salt available in Mexico City is fluoridated (200–250 mg/kg).

### 2.3. Study Group

The school’s population consisted of 350 children aged 7 to 10 years, 295 of whom provided signed informed consent (84.3%).

Before any screening, written informed consent was obtained from parents and verbal assent from the children. The exclusion criteria included children with structural enamel defects (e.g., amelogenesis imperfecta, dentinogenesis imperfecta, severe fluorosis) (n = 2), fixed orthodontic appliances (n = 12), or caries lesions and filled teeth (n = 10), or those taking central nervous system medications, such as ADHD medication (**n** = 8). A total of 32 children were excluded by these criteria. None of the participants had received antibiotics for at least three weeks prior to bacteriological sampling.

**Figure 1:**
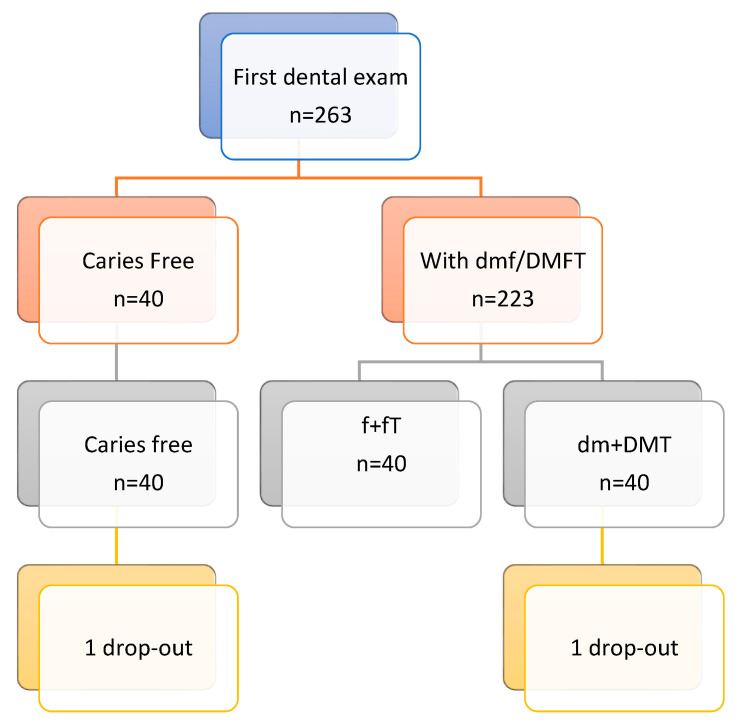
Flow chart from the child selection process.

The sample size was calculated to detect a difference in the proportion of children developing new caries lesions (yes/no) between caries-free and caries-active groups during follow-up. It was assumed that 20% of children in the caries-free group and 45% in the caries-active group would develop new lesions [5]. A power of 0.80 and an alpha of 0.05 were applied for sample size calculation. Based on this information, 40 children were required in each group (sample size calculated using Stata V17 software). There were 263 children in the full group (Figure 1).

We aimed to compare caries increment in caries-free and caries-active children. Additionally, we included a group with filled teeth but no active lesions. Of the 263 children, 50 were caries-free, and 74 had only filled teeth. The remaining 139 children had both filled and active caries lesions. A total of 120 children were followed, with 40 individuals randomly selected for each group: (1) caries free, (2) filled teeth without active caries, and (3) active caries (untreated lesions). After the baseline, two children were transferred to other schools (1.7% drop-out rate). The final sample consisted of 118 schoolchildren.

### 2.4. Questionnaire

The demographic information of the children was obtained from the parents or guardians. The children filled out a self-administered questionnaire regarding sugary food consumption between meals (chocolates, candies, cookies, snacks, soft drinks, fruit and natural juices). Also, information on oral hygiene habits (number of times the child brushes their teeth per day) was collected.

### 2.5. Caries

Baseline caries experience (dmf/DMFT) was assessed following the World Health Organization criteria [7]. Radiographs were not obtained. Two calibrated examiners carried out all the clinical examinations under natural light (intra-examiner Kappa = 0.95; inter-examiner Kappa = 0.90), following WHO recommendations. To reduce information bias, the follow-up caries assessment was conducted with the dental examiner blinded to each child’s initial caries record.

### 2.6. Caries Increment

The dental examination was repeated two years later, and the caries increment was calculated for each child using the following formula:

$${Caries\;Increment}_{i}=\sum_{t=1}^{T_{i}}I\left({lesion}_{itf}=1\;⋀\;{lesion}_{itb}=0\right)$$

Explanation of terms:

i: Index for child $\left(i=1,\;2,\;3,\ldots ,\;118\right)$.
t: Index for the tooth t.
$T_{i}$: Total number of teeth present at follow-up, for child i.
${lesion}_{itb}$: Status of tooth t at baseline $\left(0=sound,1=caries\right)$, for child i.
${lesion}_{itf}$: Status of tooth t at follow-up $\left(0=sound,1=caries\right)$, for child i.
$I\left(\cdot \right)$: Indicator function, equaling 1 if the condition inside is true (tooth developed a new lesion), and 0 if false.

### 2.7. Saliva Flow Assessment

The principal investigator (LSP) calibrated the salivary collection methods. At the beginning of the study, the unstimulated salivary flow rate was determined by asking the child to use the spitting method for 5 min. To assess the stimulated salivary flow rate, the child chewed a sterile paraffin pellet for 5 min, and the saliva produced during this time was collected in a graduated test tube. Each result is expressed in mL/min. The salivary buffer capacity was measured using Dentobuff^®^ (Orion Diagnostica, Espoo, Finland). A drop of stimulated saliva was placed on the pad of the test strip, and the result was obtained after 5 min. The buffering capacity was recorded by comparing the color of the strip with the color chart provided by the manufacturer, reported as low (pH below 4), medium (pH between 4.5 and 5.5), or high (pH over 6) [8].

### 2.8. Bacteria Levels/Counts in Saliva

*Streptococcus mutans* (*S. mutans*) counts were obtained from all children. Briefly, the child was comfortably seated on a chair, and stimulated mid-morning salivary samples were collected. Instructions were given to swallow any excess saliva, and the rough surface of the round-tipped strip was then pressed against the saliva remaining on the tongue. Bacitracin disks provided in the Dentocult^®^ SM kit (Orion Diagnostica, Espoo, Finland) had been placed in the selective culture broth 15 min before sampling. The sample-carrying strips were placed in the activated culture vials and incubated in an upright position at 37 °C for 48 h, according to the manufacturer’s instructions. After incubation, the SM colony density on the strip was assessed against a chart provided by the manufacturer.

Count ranges were categorized as follows: 0 = negative counts (NC); 1 ≤ 10^3^ CFU/mL; 2 ≤ 10^5^ CFU/mL; 3 = 10^5^ to <10^6^ CFU/mL; and 4 ≥ 10^6^ CFU/mL. SM counts were interpreted as follows: 0–10^5^ CFU/mL of saliva represented a low caries risk, and ≥10^5^ CFU/mL represented a high caries risk [9].

*Lactobacillus* counts were estimated using Dentocult^®^ LB (Orion Diagnostica, Espoo, Finland). Briefly, the child chewed a wax tablet for 5 min, and the collected saliva was poured over both sides of the test strip, draining the excess saliva. The culture broth holder was inserted into its plastic tube, closed, and then incubated at 37 °C for 4 days. The number of colonies on the test strip was compared with the chart provided by the manufacturer. The results were grouped into five categories: no growth, and 10^3^, 10^4^, 10^5^, and 10^6^ CFU/mL. Counts < 10^5^ were considered low risk, and ≥10^5^ high risk [10].

### 2.9. Statistical Analysis

The clinical, salivary, bacteriological, and behavioral markers were summarized using descriptive statistics. In the bivariate analysis of the association between quantitative variables, a Wilcoxon/Kruskal–Walli’s test with the comparison of all pairs with Dunn test analysis was used; for the qualitative variables, a chi-square analysis was applied. Subsequently, children were categorized into two groups: those who developed new caries lesions during the follow-up period and those who did not. Multiple logistic regression analyses were conducted to assess the association between caries increment (yes/no) and baseline clinical, salivary, and bacterial risk markers. Interaction terms between theoretically relevant variables were also tested. Model fit was evaluated using the Hosmer–Lemeshow goodness-of-fit test. Statistical significance was set at *p* < 0.05. All analyses were performed using STATA version 17.0 (StataCorp LLC, College Station, TX, USA).

## 3. Results

A total of 118 children aged 7 to 10 years participated in the study follow-up. The mean age was 8.3 years (SD = 1.1), and 50% of the participants were boys. Regarding oral hygiene habits, 14.4% of the children (n = 17) reported brushing their teeth once a day, about a quarter (25.4%, n = 30) brushed twice a day, and 60.2% (n = 71) brushed three times a day. All children reported using toothpaste containing fluoride.

In terms of daily sugar intake, 37% (n = 44) consumed sugary foods or meals three times per day, 40% (n = 47) four times per day, and 20% (n = 23) five times per day. The baseline means are presented in Table 1. The mean unstimulated salivary flow rate was 0.7 mL/min (SD 0.7), and the mean stimulated salivary flow rate was 1.9 mL/min (SD 0.9). Forty-two per cent of the children had high salivary buffer capacity, 38% medium, and 20% low. High bacterial counts for lactobacilli (≥10^5^ CFU/mL) were observed in 59% of the children (Table 1). The prevalence of *S. mutans* was 91.6%, and that of *Lactobacillus* sp. was 94.9%. The percentages of children with high *S. mutans* and *Lactobacillus* sp. counts were 22.0%, and 55.9%, respectively.

The distribution of risk markers by age is presented in Table 2. At baseline, not all children had deciduous teeth; six 10-year-old children had only permanent teeth. The sugar intake from foods/meals per day was higher in the seven-year-old group (*p* = 0.02). The unstimulated salivary flow rate was stable at around 0.6 mL/min, without differences by age or sex (*p* = 0.19; *p* = 0.85, respectively). The stimulated salivary flow rate increased with age, from 1.7 mL/min at seven years to 2.2 mL/min at 10 years (*p* = 0.17). It was higher in boys (2.1, SD 0.9) than in girls (1.7, SD 0.9), with significant differences (*p* = 0.01).

The results for salivary buffer capacity indicated that 42.4% (n = 50) of children had high capacity, 38.1% (n = 45) medium, and 19.5% (n = 23) low buffer capacity. No significant differences in buffer capacity by age were observed (*p* = 0.38). Additionally, no significant differences in *S. mutans* count distribution were observed by age (*p* = 0.74) or sex (*p* = 0.09). More than half of the children (56%) had high lactobacilli counts, without significant differences by age (*p* = 0.47) or sex (*p* = 0.46).

Based on caries experience, participants were divided into three groups: caries-free (n = 39), filled-teeth (n = 40), and caries-active (n = 39). The mean dental caries indices and risk markers are presented in Table 3. No significant differences by sex were found between groups (*p* = 0.80). Children in the filled-teeth group were younger than those in the other two groups (*p* = 0.0142). Most children reported brushing three times a day, with no significant differences between groups (*p* = 0.06).

After a two-year follow-up, the caries index in primary teeth decreased in all three groups. No significant differences between groups were found for sugar intake from foods/meals per day (*p* = 0.3097), stimulated salivary flow rate (*p* = 0.3759), buffer capacity (*p* = 0.2065), or bacterial counts. A significant difference was detected in unstimulated salivary flow rate, which was higher in the filled-teeth group (*p* = 0.0129) (Table 3).

The caries increment in deciduous teeth was 0.7 (SD 1.6) in the caries-free group, 0.7 (SD 1.1) in the filled-teeth group, and 0.1 (SD 0.2) in the caries-active group, with significant differences (*p* = 0.01). The caries increment in permanent dentition was 0.2 (SD 0.67), 0.2 (SD 0.36), and 0.6 (SD 1.39) in the caries-free, filled-teeth, and caries-active groups, respectively; these differences were not significant (*p* = 0.0827). The number of children who developed new caries lesions was 36 (30.5%). By group, 10 (25.6%) children in the caries-free group, 15 (37.5%) in the filled-teeth group, and 11 (28.2%) in the caries-active group developed new caries lesions, with no significant difference between groups (*p* = 0.48).

Table 4 presents the results of the logistic regression model for caries increment and the clinical, salivary, and bacteriological risk markers studied. Considering the crude and adjusted odds ratios [ORs], a crude OR in the whole analysis, and the adjusted OR is the relationship between two variables only (an exposure and an outcome). Significant variables in the model were low frequency of daily toothbrushing [OR = 2.77, 95% CI 1.126–6.825], high *S. mutans* counts [OR = 3.38, 95% CI 1.002–11.389], and high *Lactobacillus* counts [OR = 2.91, 95% CI 1.118–7.586]. Age, unstimulated salivary flow rate, and group (caries-free, filled-teeth, and caries-active) were not significant in the model.

## 4. Discussion

The results of this two-year follow-up study suggest that toothbrushing frequency, and *Lactobacillus* and *S. mutans* counts are risk markers for the increment of dental caries. Children with lower toothbrushing frequency had a higher increment of dental caries. Infrequent toothbrushing allows dental plaque to accumulate for longer periods, promoting the growth of cariogenic bacteria and acid production, which favors enamel demineralization. Several studies have shown that oral hygiene habits are associated with dental caries risk; a systematic review and meta-analysis identified higher caries increment in children with low brushing frequency in both primary and permanent dentition [11].

Longitudinal studies have also found an association between low toothbrushing frequency (<2 times per day) and caries increment [3,12]. In the Iowa Fluoride Study, a greater improvement in toothbrushing frequency was associated with lower dental caries increment. In this US study, prediction models of caries increment included sugar consumption, maternal education, daily fluoride intake, and toothbrushing frequency; only toothbrushing frequency remained significant in the multivariate models [3].

In a Brazilian cohort study, higher brushing frequency was associated with lower caries experience when comparing one to two times and two to three times per day, suggesting an advantage in brushing three times compared with two times per day [13]. A randomized clinical trial in Chinese adolescents reported that failure to improve oral hygiene habits increased the number of teeth affected by dental caries over a 24-month follow-up [14].

Consistent daily plaque removal through toothbrushing has been shown to disrupt biofilm formation, reduce bacterial load, and maintain the remineralization–demineralization balance in favor of tooth preservation [15]. However, some studies have not found an association between dental caries and toothbrushing frequency [16]. The effect of toothbrushing on dental biofilm is influenced by many factors, such as the type and condition of the toothbrush used, duration of brushing, brushing technique, and type of dentifrice. Variations in these factors may make it difficult to establish a clear relationship between toothbrushing frequency and dental caries experience.

In the present study, bacterial counts were associated with the increment of dental caries: more than one-fifth of children were exposed to high counts of *S. mutans*, and 55.9% had high *Lactobacillus* counts. High counts of these bacterial groups enhance the cariogenicity of the oral biofilm [17]. Additionally, PCR-based identification of *S. mutans* has shown this bacterium to be more prevalent in the biofilm of children with dental caries compared with caries-free children [18].

Similarly, a literature review considering both bacterial types in saliva found that mutans streptococci and lactobacilli were more frequently detected in caries-active children and adolescents than in caries-free peers [19]. Moreover, the salivary microbiome is often more diverse and richer in caries-active individuals [20]. A study of the microbiome of Korean children aged 6–12 years found that caries-active children had high counts of *S. mutans* [21], while caries-free children harbored more *Corynebacterium*. The authors suggested that these bacterial differences and age could be used as caries risk markers [21,22].

Using modern technology, other bacteria have also been identified as associated with caries increment, such as *Candida*, *Fusobacterium*, *Prevotella*, and others [18,23]. Further studies are needed to clarify the interactions between lactobacilli, *S. mutans*, and other bacteria in the oral biofilm and their role in dental caries risk assessment.

It is very important to develop a structured school program in Mexico City that actively involves dentists and teachers to improve oral health in our children, as well as to emphasize the importance of implementing risk stratification, supported by microbiological screening, especially for individuals identified as high risk.

Children with active caries tend to acquire cariogenic microbiomes at an earlier age than their caries-free peers. As children grow older, particularly during the mixed dentition stage, the prevalence of infection increases, and most individuals harbor cariogenic bacteria [22]. Advances in understanding the oral microbiome suggest that the balance between protective and pathological factors must be considered when assessing caries risk. This balance involves strategies to stabilize oral pH and limit the consumption of sugary foods, particularly in children [6,24,25].

In the present study, children who were caries-free at baseline had the lowest mean dental caries index at the end of the two-year follow-up compared with those in the filled-teeth and caries-active groups. However, the caries-free group did not differ significantly from the other two groups in the development of new caries lesions, and baseline caries experience was not significant in the multivariate model. Similar findings have been reported in studies in the USA and Japan, where a large proportion of new caries lesions occurred in children who were caries-free at baseline [26,27].

The lack of differences in caries increment among the three groups may be related to the low number of children who developed new cavitated lesions, as fewer than one-third presented new lesions during the two-year follow-up. In addition, all participants received dental education sessions, which may have promoted improved oral health practices across groups and reduced the likelihood of detecting between-group differences. Longer follow-up periods may be necessary to detect differences in caries progression, particularly given the widespread availability of fluoride sources in Mexico, including the national salt fluoridation program and the common use of fluoridated dentifrices. Additionally, at baseline, no differences were observed in the frequency of high sugary food consumption between the groups; if dietary habits were comparable, the risk of developing new caries lesions may also have been similar.

Dental caries is a cumulative condition shaped by early-life exposures, many of which may not be captured by baseline dietary or salivary assessments. Its development is multifactorial, influenced not only by diet, bacterial composition, and saliva, but also by host factors such as tooth morphology, which may help explain the lower caries experience observed in some children at baseline and during follow-up. Caries-active children are likely to acquire cariogenic infection at an earlier age than caries-free peers, giving them a head start in the disease process. However, as children grow older, particularly during the mixed dentition stage, the opportunity for exposure increases, and differences between groups diminish as most children eventually harbor cariogenic bacteria [22,28].

With the information we have presented, it is very important to raise awareness among parents, dentists, and teachers about the importance of introducing knowledge about oral health and preventive methods from the primary dentition, the transition to mixed dentition, and the importance of awareness about oral hygiene at this stage of child development.

Caries risk assessment has the potential to improve patient care, as it is fundamental to developing an appropriate treatment plan. Such planning should account for risk factors associated with socioeconomic status, geographic region, and the preventive measures adopted at the national level [29]. In this context, the highly skewed distribution of caries prevalence highlights the need for new and more effective preventive strategies beyond fluoride, particularly for individuals at high risk [30].

This study has some limitations. Although each study group met the required sample size determined by our a priori calculation, the overall sample is not representative of all schoolchildren in Mexico City. Nevertheless, the socioeconomic background and access to dental care of the study participants are comparable to those of most children attending public schools in the city, supporting the applicability of our findings to similar populations.

In addition, self-reported information from children on sugar and soft drink consumption may be imprecise, and data on the intake of other foods in their diet were not collected. Studying also has several strengths. It included a two-year follow-up and assessed salivary, bacterial, and behavioral factors commonly associated with dental caries. All the children receive oral health education and new toothbrushes every six months; this can change exposure and dampen increment differences. These results highlight the multifactorial nature of the condition and underscore the importance of identifying reliable markers to detect children at high risk of dental caries.

It is essential to emphasize that early education involving all actors in the oral health–disease process (children, parents, teachers, dentists, and health systems) is crucial to establishing the foundation for knowledge and awareness, constituting one of the most important preventive strategies for the oral health of our population.

## 5. Conclusions

Dental caries prevalence was high in the study group, particularly in the primary dentition. Although differences in new caries lesions among children with different baseline caries experiences were not statistically significant, daily toothbrushing and salivary counts of *S. mutans* and *Lactobacillus* were identified as risk markers for caries increment. These findings emphasize the multifactorial nature of dental caries and the importance of maintaining effective oral hygiene and monitoring cariogenic bacteria to prevent disease progression in school-aged children.

## Figures and Tables

**Table 1:** Baseline characteristics of the study population.

Clinical Risk Markers	Median	Mean (SD)
Age	8	8.3 (1.1)
Sugar intake in foods/meals per day	4	3.8 (0.8)
Daily brushing (F^−^ toothpaste)	3	2.4 (0.8)
dmft	6.5	4.8 (3.3)
DMFT	0	0.6 (1.1)
dmf/DMFT	7	5.1 (3.9)
**Salivary risk markers**	n	**%**
Unstimulated salivary flow rate (mL/min)	0.5	0.7 (0.7)
Stimulated salivary flow rate (mL/min)	2.0	1.9 (0.9)
Buffer capacity	**n**	**%**
High	50	42.4
Medium	45	38.1
Low	23	19.5
**Bacterial risk markers**	**n**	**%**
*S. mutans*		
<10^5^	92	77.9
≥10^5^	26	22.0
*Lactobacillus* sp.		
<10^5^	48	40.6
≥10^5^	70	59.3

(SD) = Standard Deviation; dmft: mean of decayed, missing, and filled primary teeth; DMFT: mean of decayed, missing, and filled permanent teeth; mL/min = milliliters per minute.

**Table 2:** Dental caries risk markers by age at baseline.

Clinical Risk Markers	Years-Old				
	7 n = 31	8 n = 38	9 n = 29	10 n = 20	
	Mean (SD)	Mean (SD)	Mean (SD)	Mean (SD)	*p* value *
Sugar intake foods/meals per day	4.1 (0.8) ^a^	3.6 (0.8) ^ab^	3.5 (0.6) ^b^	3.9 (0.9) ^ab^	0.0238
Daily brushing	2.5 (0.7)	2.5 (0.6)	2.5 (0.8)	2.3 (0.9)	0.5801
dmft	4.6 (3.3)	5.1 (3.4)	4.3 (3.4)	5.1 (3.4)	0.8125
DMFT	0.3 (0.8)	0.5 (1.1)	0.9 (1.2)	0.6 (1.2)	0.2108
dmf/DMFT	4.9 (3.2)	5.6 (3.7)	5.2 (4.2)	4.2 (4.4)	0.6343
**Salivary risk markers**					
Unstimulated salivary flow rate (mL/min)	0.6 (0.3)	0.8 (1.1)	0.6 (0.5)	0.6 (0.4)	0.1949
Stimulated salivary flow rate (mL/min)	1.7 (0.7)	1.8 (0.8)	1.9 (0.9)	2.2 (1.1)	0.3159
Buffer capacity	**%**	**%**	**%**	**%**	*p* value **
High	9.3	11.0	13.6	8.5	0.3785
Medium	9.3	15.3	8.5	5.1	
Low	7.6	5.9	2.6	3.4	
**Bacterial risk markers**					*p* value *
*S. mutans*	**%**	**%**	**%**	**%**	
<10^5^	22.0	24.6	17.8	13.6	0.7391
≥10^5^	4.2	7.6	6.9	3.4	
*Lactobacillus* sp.					
<10^5^	8.4	16.1	11.9	7.6	0.4731
≥10^5^	17.8	16.1	12.7	9.3	

(SD) = standard deviation; * *p* value using Wilcoxon/Kruskal–Wallis test with comparison of all pairs with Dunn test; ** *p* value using Chi^2^ test; means with the same letters are not statistically significantly different from one another; dmft: mean of decayed, missing, and filled primary teeth; DMFT: mean of decayed, missing, and filled permanent teeth; mL/min = milliliters per minute; % = percentage of children with risk factor.

**Table 3:** Risk variable distribution by group.

Caries Risk Variables	Caries Free n = 39	Filled Teeth n = 40	Caries Active n = 39	*p* Value *
	Mean/SD	Mean/SD	Mean/SD	
Age	8.5 (1.1) ^a^	7.9 (0.9) ^b^	8.5 (1.0) ^a^	<0.0142
Sugar intake foods/meals per day	3.6 (0.8) ^a^	3.9 (0.7) ^a^	3.8 (0.9) ^a^	0.3097
Daily brushing	2.4 (0.8) ^a^	2.7 (0.5) ^a^	2.3 (0.8) ^a^	0.0588
dmft _initial_	0 ^c^	6.2 (1.8) ^b^	7.4 (0.5) ^a^	<0.0001
dmft _final_	0.7 (1.5) ^b^	5.1 (1.9) ^a^	5.2 (2.1) ^a^	<0.0001
DMFT _initial_	0 ^b^	0.8 (1.2) ^a^	0.9 (1.3) ^a^	<0.0004
DMFT _final_	0.2 (0.7) ^b^	1.0 (1.3) ^a^	1.4 (0.3) ^a^	<0.0001
dmf/DMFT _Initial_	0 ^c^	7.0 (2.3) ^b^	8.2 (1.4) ^a^	<0.0001
dmf/DMFT ^Final^	0.6 (1.4) ^b^	5.2 (2.5) ^a^	4.5 (2.9) ^a^	<0.0001
DMFT increment	0.2 (0.7) ^a^	0.2 (0.4) ^a^	0.6 (1.4) ^a^	0.0827
**Salivary risk markers**				
Unstimulated salivary flow rate (mL/min)	0.6 (0.4) ^a^	0.8 (1.1) ^ab^	0.6 (0.3) ^a^	0.0129
Stimulated salivary flow rate (mL/min)	2.0 (0.9) ^a^	1.9 (0.9) ^a^	1.7 (0.8) ^a^	0.3759
Buffer capacity	**%**	**%**	**%**	*p* value **
High	17.8	13.6	11.2	0.2065
Medium	9.3	11.9	17.0	
Low	5.9	8.5	5.1	
**Bacterial risk markers**				
*S. mutans*	**%**	**%**	**%**	
<10^5^	28.0	23.7	26.8	0.2817
≥10^5^	5.1	10.2	6.8	
*Lactobacillus* sp.				
<10^5^	17.8	15.3	11.0	0.1873
≥10^5^	15.4	18.6	23.1	

SD = standard deviation; dmft = mean of decayed, missing, and filled primary teeth; DMFT = mean of decayed, missing, and filled permanent teeth. * *p* value using Wilcoxon/Kruskal–Walli’s test with comparison of all pairs with Dunn test; means with the same letters are not statistically significantly different from one another; ** *p* value using Chi^2^ test.

**Table 4:** Crude and adjusted odds ratios for predictors of dental caries increment.

Variable	Crude OR [95% CI]	Crude *p*-Value	Adjusted OR [95% CI]	Adjusted *p*-Value
Sugar intake in foods/meals per day ^(1)^	0.87 [0.54–1.43]	0.592	0.70 [0.40–1.21]	0.201
Daily toothbrushing ^(2)^	2.28 [1.02–5.07]	0.044	2.77 [1.13–6.83]	0.026
Unstimulated salivary flow rate ^(3)^	0.75 [0.33–1.71]	0.489	0.61 [0.19–1.96]	0.409
*S. mutans* ^(4)^	2.15 [0.80–5.77]	0.128	3.38 [1.01–11.39]	0.050
*Lactobacillus* sp. ^(5)^	2.27 [0.99–5.22]	0.053	2.91 [1.12–7.59]	0.029
Age	0.78 [0.47–1.07]	0.486	0.73 [0.47–1.13]	0.153
Group ^(6)^				
Filled teeth	1.74 [0.67–4.56]	0.259	1.39 [0.43–4.46]	0.578
Caries active	1.14 [0.42–3.10]	0.799	0.78 [0.25–2.39]	0.658

Cut-off points: sugar intake three or more times per day ^(1)^; toothbrushing once or less per day ^(2)^; salivary flow rate ≤ 0.5 mL/min ^(3)^; *S mutans counts ≤* 10^5 (4)^; *Lactobacillus* sp. *counts ≤* 10^5 (5)^; group: caries-free ^(6)^; model fit: Hosmer–Lemeshow *p* > 0.05.

## Data Availability

The data presented in this study are available on request from the corresponding author. The data are not publicly available due to privacy or ethical restrictions. The informed consent signed by the parents includes this restriction.

## Abbreviations

The following abbreviations are used in this manuscript:

DCBS	Division de Ciencias Biologicas y de la Salud
ADHD medication	medications used to treat Attention-Deficit/Hyperactivity Disorder.
dmft	mean of decayed, missing and filled primary teeth
DMFT	means of decayed, missing and filled permanent teeth
pH	potential hydrogenated
SD	standard deviation
mL/min	milliliters per minute;
dmft/DMFT	addition of mean of decayed, missing and filled primary teeth and mean of decayed, missing and filled permanent teeth
OR	odds ratio
